# Detecting Malware with Information Complexity

**DOI:** 10.3390/e22050575

**Published:** 2020-05-20

**Authors:** Nadia Alshahwan, Earl T. Barr, David Clark, George Danezis, Héctor D. Menéndez

**Affiliations:** 1 Computer Science Department, University College London, London WC1E 6BT, UK; nadia.alshahwan@ucl.ac.uk (N.A.); e.barr@ucl.ac.uk (E.T.B.); david.clark@ucl.ac.uk (D.C.); g.danezis@ucl.ac.uk (G.D.); 2 Computer Science Department, Middlesex University London, London NW4 4BG, UK

**Keywords:** information theory, Kolmogorov complexity, normalized compression distance, malware detection

## Abstract

Malware concealment is the predominant strategy for malware propagation. Black hats create variants of malware based on polymorphism and metamorphism. Malware variants, by definition, share some information. Although the concealment strategy alters this information, there are still patterns on the software. Given a zoo of labelled malware and benign-ware, we ask whether a suspect program is more similar to our malware or to our benign-ware. Normalized Compression Distance (NCD) is a generic metric that measures the shared information content of two strings. This measure opens a new front in the malware arms race, one where the countermeasures promise to be more costly for malware writers, who must now obfuscate patterns as strings qua strings, without reference to execution, in their variants. Our approach classifies disk-resident malware with 97.4% accuracy and a false positive rate of 3%. We demonstrate that its accuracy can be improved by combining NCD with the compressibility rates of executables using decision forests, paving the way for future improvements. We demonstrate that malware reported within a narrow time frame of a few days is more homogeneous than malware reported over two years, but that our method still classifies the latter with 95.2% accuracy and a 5% false positive rate. Due to its use of compression, the time and computation cost of our method is nontrivial. We show that simple approximation techniques can improve its running time by up to 63%. We compare our results to the results of applying the 59 anti-malware programs used on the VirusTotal website to our malware. Our approach outperforms each one used alone and matches that of all of them used collectively.

## 1. Introduction

Arms races are often ruinous. The malware arms race is no exception. Despite the widespread use of antivirus software, malware is imposing a significant productivity tax on society, slowing machines and wasting bandwidth. Moreover, there are no SALT talks to provide respite periods. The race is relentless.

The evolving sociology of malware, and in particular the growth of industrial-scale production of polymorphic and metamorphic variants of existing malware, is straining the ability of existing methods of detection, via signatures and dynamic analysis, to cope with this production volume. A number of researchers have considered ways to leverage existing techniques. Since the early to mid 2000s, there has been research into semantics of programs, on the assumption that identifying semantic invariants can help to overcome metamorphic variation.

This decade has seen research into similarity metrics and applications of machine learning. The motivation is that signature-based approaches, whether syntactic or behavioural, cannot easily handle previously unseen malware. In contrast, a successful similarity metric could extrapolate the features of its two labelled input sets of malware and non-malware and, at least some of the time, avoid upfront analysis and detect previously unknown malware, automatically.

A universal, generic similarity metric has existed for over a decade, the Normalised Information Distance (NID) [1]. It works on syntax rather than semantics, but it has a deep mathematical foundation that is connected to information theory, probability theory, the theory of randomness, and algorithmic complexity theory. It has the unparalleled advantage of being universal in the sense that it minorises (intuitively: incorporates) every other possible similarity measure, but has the slight drawback of being uncomputable. Fortunately, an effective computable approximation to NID exists that uses compressors, the Normalised Compression Distance (NCD) [2].

The promise of building a malware detector on the back of NCD is its good approximation to universality. If NCD detects malware well, it could do so without requiring any static or dynamic analysis or preprocessing. All one would need to detect malware is sufficient processing power, a good compressor, and labelled collections of malware and benign-ware. Being generic, it can be directly applied to binary executables. Its use for detecting malware promises to open a new front in the malware arms race, one in which the advantage lies with the malware detector, because it increases the cost of producing variants that remain variants, i.e., preserve malware behaviour, but share less information, universally construed, at the level of strings.

Our objective in this paper is to answer the question, “How well does NCD detect malware when we apply it to binary executables?”, and to answer it with empirical rigour. The answer, incidentally, is, “Very well!”. We found that when you apply the method to malware collected within a short time scale of a few days, it detects malware with 97.4% accuracy; rather astonishing from a standing start. To attain this 97.4% level, we augmented our technique with compressibility rates and combined these with NCD values using a decision forest classifier. This shows that our technique is amenable to improvement.

This paper makes the following contributions:

1. It conducts the first statistically rigorous, experimental evaluation of the ability of NCD to detect malware using only binary executables.
2. It demonstrates that NCD, as used in our approach, is competitive with commercial anti-malware tools: it outperforms any single one and matches the performance of all of them together.
3. It shows that NCD complements existing, state-of-the-art classifiers, by combining it with the decision forest classifier, which is inherently parallel and tunable.

We were inspired by Wehner’s 2007 paper in which she applied NCD to cluster polymorphic worms found in network traffic [3]. Our work builds on her observational descriptive statistics to build and rigorously evaluate an NCD-based malware classifier.

## 2. Background

This paper is founded on the use of compressors to achieve upper bounds on the Kolmogorov complexity (also called algorithmic information and information complexity) of strings. Our interest is in detecting malware through the application of a universal, generic, similarity metric called the Normalised Information Distance (NID).

Let us unpack some aspects of this description of the metric. First, it is a distance metric and satisfies the axioms of non-negativity, the identity of indiscernibles, symmetry, and the triangle inequality. Second, it is generic in the sense that it can be applied to any type of string: genomes, binary executables, JavaScript programs, MP3s, etc. Third, it is normalised so that strings of different length can be compared, and the resulting measure is always in the unit interval, [0,1]. Fourth, it is a universal similarity metric based on information complexity.

The universality of the metric is wherein much of its power lies and is particularly interesting from the perspective of malware detection. The information complexity of a string is essentially a measure of the randomness of the string, and the information distance between two strings is a measure of their relative randomness. Randomness is a very pure thing: the absence of a pattern. It helps the intuition about the similarity metric to consider that, by measuring relative randomness, one also measures its opposite: all possible patterns in common. This is the intuition that underlies the universality of the information distance. If there is a pattern in common between two strings, NID will detect it and measure it. Mathematically, this appears as a minorisation property of the metric. Because it detects all patterns, it produces a distance shorter than any other similarity measure does because it subsumes that measure. This universality makes it very difficult to overcome in an adversarial situation. How does a malware developer improve the relative randomness of each new malware with respect to all existing ones? Certainly not through automated methods.

Fortunately for the malware writer, NID, like Kolmogorov complexity, is not computable, and we are forced to use the best, general approximation to it, the Normalised Compression Distance (NCD). On the other hand, compressors are themselves quite general pattern detectors, and the more effective they are, the better the length of the compressed string approaches the Kolmogorov complexity of the original. Malware authors are left with finding anti-patterns for all and any existing compression techniques.

In what follows, we outline the underlying ideas and provide intuitions about how the similarity metric works, and we outline the relationship between Kolmogorov complexity and NCD. More detail may be found in the papers by Li, Cilibrasi, Vitányi, and others and in Li and Vitanyi’s book [1,4,5].

First, we work in a world of strings so any objects we wish to consider must be encoded as strings. This is not a strong restriction, as numbers, computer programs, and many other objects can be encoded as strings. Therefore, encode all objects as binary strings $x\in {\left\{0,1\right\}}^{*}$. We can totally order all such strings, first according to length, then lexicographically within each length. Every string in the ordering, x, can then be identified with the index of its position in the ordering, where its length is:

$$\begin{array}{c} \mathrm{len}(x)=\lfloor \log(x+1)\rfloor . \end{array}$$

It is desirable that we can work, if possible, in a setting where the set of strings we consider is a prefix set, i.e., no string in the set is the proper prefix of another. This restriction is not necessary, but desirable. The prefix set property tends to imply other good properties, for example being able to associate a probability distribution (technically a semi-measure) with binary strings. One way to obtain a prefix-free encoding of numbers and programs is to use a self-delimiting code for a string, *x*, such as:

$$\begin{array}{c} \bar{x}=1^{\mathrm{len}(x)}0x \end{array}$$

Then, it can be shown that $\left\{\bar{x}:x\in {\left\{0,1\right\}}^{*}\right\}$ is prefix-free [4]. Any programming language with an end program marker is prefix free, including Java, Lisp, and programs for the universal Turing machine.

Kolmogorov complexity is sometimes called the universal minimum description length. The act of description requires a description language, so we say that φ(p)=x means that *p* is a description of *x* using the description language φ. Now, we can define the conditional complexity of a string given a starting string as the length of the shortest description that takes the given starting string as input and produces the string.

**Definition** **1** (The conditional complexity of a string)**.** *The conditional complexity of x given y is:*

$$C_{\varphi }\left(x|y\right)=min\left\{len\left(p\right):\varphi \left(y,p\right)=x\right\}$$

Here, *p* is a partial recursive function that takes *y* as input and outputs *x*. The conditional complexity of *x* is just the length of the shortest function that can do this.

It can be shown that the set of partial recursive functions on a domain of prefix-free strings is sufficiently expressive to capture description languages and that, for every pair of strings, there is, in theory, a best description language in this set in that descriptions in this language are shorter than descriptions in any other language for this pair of strings.

**Theorem** **1.** ∃ *a partial recursive prefix function* $\Psi_{0}$ *s.t.* ∀ *partial recursive prefix functions* Ψ, *there is a constant c with:*

$$C_{\Psi_{0}}\left(x|y\right)\leq C_{\Psi }\left(x|y\right)+c$$

*for all pairs of strings x and y. The constant c is independent of x and y, only depending on $\Psi_{0}$ and* Ψ.

The importance of this theorem is that minimum description length is language dependent, but by fixing the description language, we do not lose out. As we consider increasingly longer strings, the concept of a minimal description length independent of the description language asserts itself. One consequence is that when we apply our similarity metric to malware, confidence in our results increases with the length of the strings compared.

The conditional Kolmogorov complexity of a string *x* given a string *y* is written $K\left(x|y\right)=C_{\Psi_{0}}\left(x|y\right)$, while the (unconditional) Kolmogorov complexity of a string is written $K\left(x\right)=C_{\Psi_{0}}\left(x|\epsilon \right)$, and $\Psi_{0}$ is usually taken to be a universal computer such as a particular optimal universal Turing machine. An “optimal” universal effective mapping has the type of universality such that each image of a string has a length that is up to a constant shorter than what can be achieved by another particular universal mapping of all strings.

The similarity metric ideally should be a distance metric, i.e., should satisfy the axioms of a distance metric. The obvious definition for the minimal information distance between a pair of strings, *x* and *y*, is the length of the shortest program for a universal computer to transform *x* into *y* and *y* into *x*. However, the conditional algorithmic complexities of two strings considered in each order are not, in general, equal, i.e., K(x|y)≠K(y|x). Because symmetry fails, this “obvious” definition is not a distance metric. This can be fixed by simply taking the least upper bound of the conditional complexities. Then, there remains the problem of comparing strings of quite different lengths. “Normalising” the distance with respect to the least upper bound of the algorithmic complexities of the two strings handles this problem. The result is the Normalised Information Distance (NID).

**Definition** **2** (NID)**.**

$$\begin{array}{c} e\left(x,y\right)=\frac{max\{K(x|y),K(y|x)\}}{max\{K(x),K(y)\}} \end{array}$$

NID calculates a value in [0,1] and is a universal, generic, upper semi-computable distance metric satisfying a density requirement in ${\left\{0,1\right\}}^{*}$. It is universal because it can be shown to be less than any other similarity metric between two strings. Therefore, if any two strings are similar because of any feature that they share and this can be captured in a metric, it can also be captured with NID. This universality, in turn, makes NID completely generic. It does not depend on any particular features of the strings, so it can be applied to any type of strings. It is upper semi-computable because it can be approximated from above by a sequence of functions into the rational numbers that converge on NID in the limit. It is a useful, non-degenerate metric because at any finite distance from a string, there is, at most, a certain, finite number of other strings.

However, Kolmogorov complexity is not a partial recursive function, so it is not computable and must be approximated. Any approximation is necessarily an upper bound, as explained above. We use compression programs to calculate computable upper bounds. Compressors are not the only possible way to approximate algorithmic information content, but they are natural, since they exploit repetitive patterns in a string. The better your compression program, the more tightly your upper bound approximates Kolmogorov complexity. Useful detection and classification methods for malware using algorithmic complexity give an advantage in the arms race as doing better may require the intellectually prohibitive cost of developing a better compressor. Even then, it is not immediately clear how this advantage can be exploited.

We follow Cilibrasi and Vitanyi in simply replacing K(x) and with Z(x) where Z(x) is the length of the compressed version of string *x* produced by a compression program *Z*. Simply substituting *Z* for *K* in the definition of NID creates the problem of interpreting Z(x|y). To sidestep this problem, Cilibrasi and Vitanyi employed the following result.

**Lemma** **1.** $$\begin{array}{c} max\{K(x|y),K(y|x)\}=K(xy)-min\{K(x),K(y)\} \end{array}$$

*where xy is the concatenation of the strings x and y.*

The result of replacing *K* with the upper bound *Z* is called the normalised compression distance.

**Definition** **3** (NCD)**.** *The normalised compression distance is given by:*

$$\begin{array}{c} e_{Z}\left(x,y\right)=\frac{Z(xy)-min\{Z(x),Z(y)\}}{max\{Z(x),Z(y)\}}. \end{array}$$

Like NID, NCD calculates a value in [0,1], although this outcome depends on how normal the compressor used is. The key term in the definition is the term Z(xy), as this is what makes the distance work. It is also the term that is more expensive to compute than *x* and *y* as it is a longer string to compress. A normal compressor is so called because it is well behaved with respect to this term.

**Definition** **4.** *Normal compressor A compressor, Z, is normal if it satisfies for all strings x, y, and z:*

1. *Z(xx)=Z(x) and Z(ϵ)=0,*
2. *Z(xy)≥Z(x),*
3. *Z(xy)=Z(yx),*
4. *Z(xy)+Z(z)≤Z(xz)+Z(yz),*

*up to an additive O(logn) term with n the maximal binary length of any string involved in the (in)equality.*

Assuming that we use a normal compressor, intuitions about how the distance works can be gained by applying NCD to a single file. Z(xx)=Z(x), so NCD(x,x)=Z(x)−Z(x)/Z(x)=0, i.e., every file is completely similar to itself. Not all compressors behave in this normal way. We discuss our choice of 7-Zip as the compressor in Section 3.

To build your intuition about how NCD works, it is instructive to consider $NCD_{S}$, which replaces the normal compressor *Z* with the file size in the definition of NCD. ${\mathrm{NCD}}_{S}$ has the unwanted property that ${\mathrm{NCD}}_{S}\left(x,y\right)=1$, for all *x* and *y*. WLOG, let S(x)<S(y), then:

$$\begin{array}{cc} NCD_{S}\;= & \frac{S(xy)-\min(S(x),S(y))}{\max(S(x),S(y))}=\frac{S(x)+S(y)-S(x)}{S(y)}\;=\;\frac{S(y)}{S(y)}\;=\;1. \end{array}$$

## 3. Classifying Malware Using NCD

The Normalised Compression Distance (NCD) works by finding common patterns between two strings using compression algorithms. Therefore, there are no restrictions on the type of strings to which we can successfully apply NCD. If we want to use NCD to find similarities between programs, we can apply it to any string representation of a program, such as the source code, an execution trace, an abstraction of the program, or the program’s binary.

Applying NCD directly to the binary representation of the program eliminates the manual effort needed to reverse engineer or execute the program to obtain an execution trace. In the case of malware, this is particularly useful because malware writers go to great lengths to prevent their programs from being reverse engineered or revealing their malicious behaviour when executed within a controlled environment like a virtual machine. Further, executing malware outside of a controlled environment is unsafe.

### 3.1. Choice of Compressor

**Figure 1:**
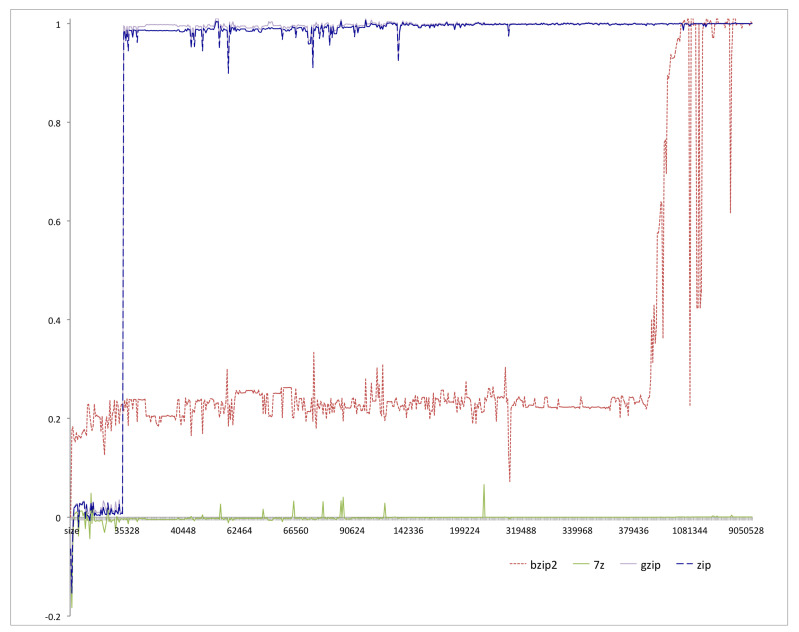
The Normalized Compression Distance (NCD) of files to themselves for different file sizes using different compressors.

NCD is an upper bound on information distance. The choice of compressor determines how tight this upper bound will be. Previous research [6] found that the size of strings we want to compare and the size of the block or window that the compressor uses affect the values of NCD. Our own experiments confirmed this finding and indicated that a compressor similar to 7-Zip performed well (i.e., using 7-zip, NCD(x,x) was close to zero) for our domain, classifying malware and benign-ware. Figure 1 shows the results of our experiment. The *x*-axis is the size of the file, while the *y*-axis is the NCD of the file to itself. We can clearly see that 7-Zip outperformed the other three compressors (gzip, winzip, and bzip2). The window size in 7-Zip can be set to a maximum of 4GB, making it suitable to calculate NCD for two files with a combined size of up to 4GB.

### 3.2. Classifier

We built a classifier based on NCD to classify individual programs. We designed a random forest classifier using NCD and compressibility ratio features to classify programs as benign or malicious. A decision forest [7] is an ensemble classifier composed of multiple, independent decision trees. Trees are trained independently over a randomly selected subset of the features at each decision point. When classifying a program, each tree’s decision is a vote; the forest’s decision is affirmative when the fraction of the affirmative votes of its trees exceeds a threshold. Tuning the decision threshold leads to classifiers with different true positive rate versus false positive rate trade-offs. Random forest training and classifying can be parallelized, and they exhibit good classification and generalization performance [8].

Feature selection: Our classifier takes as input a vector of n+1 real-valued features in the range [0.0,1.0]. We form this vector as follows. The first feature is a program’s compressibility ratio, the ratio of its compressed size to its uncompressed size. The other *n* features are the NCD of the program with *n* other reference programs, $\frac{n}{2}$ benign ones and $\frac{n}{2}$ malicious ones. We select these *n* reference programs uniformly at random from our collection of labelled programs. We can show that some features are more important to the forest’s decision than others. In future work, we plan to extract, then exploit this knowledge to choose features more effectively. The binary features used in the decisions in the decision trees in the random forest are random thresholds on one of the n+1 features.

Training: To train the random forest classifier, we start from a set of programs labelled as benign or malicious. The set of training programs is disjoint from the reference programs used to compute the NCD and from the evaluation set used subsequently to estimate the classification performance of our approach.

Training a random forest involves training each decision tree independently, while injecting sufficient randomization, as described below, to ensure robust learning. Our approach is to use the training set to train each forest, but randomize the features available to train each decision point in each tree. We defined this restricted feature set by picking an index in the feature vector uniformly at random; then, to make the decision binary, we picked a threshold, again uniformly, from all the vectors in the test set at this index.

We greedily built individual decision trees by selecting and storing a feature at each decision point, out of the restricted set, to minimize the Shannon entropy over the labels of their leaves. Concretely, given an initial set of items *B* at a decision point *d*, we tried to partition *B* into *L* over *d*’s left subtree and *R* over *d*’s right subtree to maximize the information gain:

(1) $$\begin{array}{c} I=H\left(B\right)-\left(\frac{\left|L\right|}{\left|B\right|}H\left(L\right)+\frac{\left|R\right|}{\left|B\right|}H\left(R\right)\right), \end{array}$$

where *H* denotes the Shannon entropy function over the distribution of labels in the set and |·| the cardinality of the set.

Trees are grown to their maximum height, unless no proposed feature allows a significant information gain, set by a cut-off threshold. In each decision tree, each leaf stores the number of benign and malicious programs assigned to it.

The distribution of the training data plays a role in the accuracy of the resulting classifier when applied to unknown items. The relative prevalence of labels in the training set influences the maximization of information gain, as seen in Equation (1). Increasing the prevalence of items with a particular label in the training set introduces a higher penalty for misclassifying those items. We therefore tested our classifiers under different training conditions to ensure they were robust and detected good training distributions to improve their performance.

Our training implementation was parallelized to use an arbitrary number of cores on a single computer and could easily be ported to a distributed setting.

Classification: Given an unknown program, we wish to label it as benign or malicious. First, its n+1 feature vector is computed by calculating its compressibility ratio and its NCD to the *n* reference programs. Then, each decision tree in the forest assigns the program to a leaf: decisions based on the program’s feature vector branch left or right, until a leaf is reached. We interpret each leaf as an empirical probability the item is benign or malicious, and the decisions of all trees are averaged to derive the overall likelihood for each category.

Evaluation metrics: The traditional metric for success for a classifier is accuracy, defined as the number of correctly classified items over the total number of items. Unfortunately, this measure confounds Type I and Type II errors. Considering the classifier’s false positive and false negative rates separately is easier to understand and interpret.

Since the output of a random forest is a real in [0.0,1.0], a decision boundary may be set within this range over which one classifies a program as malicious. Different decision thresholds exhibit different True Positive (TP) and False Positive (FP) rates. The receiver operating characteristic illustrates the trade-off between TP and FP for all possible decision boundaries.

The concrete problem of malware detection is one where the base rate of positives (malware) may be significantly lower than 50%. Thus, a key metric of success is the true positive rate (the fraction of positives classified as positives), for extremely low rates of false positives (the fraction of negatives misclassified over all negatives). A high rate of false negatives would otherwise lead to the vast majority of items recognized as positives being misclassifications. For this reason, our evaluation illustrates ROC curves in the false positive range of 0–10% only.

The choice of feature and training sets impacts the effectiveness of the classifier. Refining the selection process of those two sets and adding lightweight preprocessing steps (e.g., unpacking packed malware) are expected to improve the results. In this paper, however, we wanted to isolate and investigate the performance of NCD and decision forests without the interference of other techniques.

Finally, some of the n+1 features may be more important than others in providing information to classify programs as benign or malicious. To determine feature importance, we follow the approach suggested by Breiman [7] and report the fraction of trees that use a specific feature. A feature is used in a decision tree if a threshold was applied to it in a tree to decide the outcome of a branch.

### 3.3. Lower Bound on NCD

Clustering data using NCD can be time consuming because it is a pairwise measure. The most expensive part of the computation is the compression of the concatenation of the two strings. Compressing each string separately is also time consuming, but only needs to be performed once for each string while the compression of the concatenation needs to be performed for every possible combination of strings.

We can improve the scalability of using NCD if we can find a way to minimise the number of comparisons we have to make. One way to achieve this is to approximate the lowest NCD we can obtain for a pair of strings. If this value is high (for example, 0.8 or 0.9), then we know that the two strings are not similar, and we can skip calculating their NCD. We now show how to compute such a lower bound, using only the compression of each string. Assuming *Z* is a normal compressor (Section 2) and that Z(y)≤Z(x) (the case Z(y)≤Z(y) is symmetric), we have:

$$\begin{array}{cc} e_{Z}\left(x,y\right) & =\frac{Z(xy)-\min\{Z(x),Z(y)\}}{\max\{Z(x),Z(y)\}}\\ \; & =\frac{Z(xy)-Z(y)}{Z(x)}. \end{array}$$

The smallest NCD value we can then obtain occurs when *x*, the longer string, fully contains *y*, the shorter string. In this case, Z(xy)=Z(x), and we have:

$$\begin{array}{c} e_{Z}min\left(x,y\right)=\frac{Z(x)-Z(y)}{Z(x)}=1-\frac{Z(y)}{Z(x)}. \end{array}$$

For example, if Z(x)=10 and Z(y)=3, we know, without compressing xy, that the minimum NCD we can obtain is $0.7=1-\frac{3}{10}$. We can then choose a threshold, based on application domain and compute $e_{Z}min\left(x,y\right)$. If $e_{Z}min\left(x,y\right)$ exceeds this threshold, we are not interested in, and do not compute, the exact NCD(x,y).

### 3.4. Evading NCD

Large-scale production of malware depends on the automated generation of variants of the same malware. To evade detection by NCD, variant generators must increase the NCD between the original malware and its variants. To this end, they have two choices: add new content or obfuscate.

If we apply the previous formula for NCD lower bounds to a file and a variant of that file, we have:

$$\begin{array}{c} e_{Z}min\left(x,x^{\prime }\right)=1-\frac{Z(x)}{Z(x^{\prime })}. \end{array}$$

We can clearly see that we need to add at least 100% more unique content, as through obfuscation or junk injection, to $x^{\prime }$, to increase $Z(x^{\prime })$ by 100%, to increase NCD to 0.5. This NCD value might not even be enough to evade detection, as a 0.5 similarity to known malware may still flag the new variant as suspicious to an NCD-based classifier.

As we also point out in Section 2, an NCD-based classifier raises the bar for malware writers and makes generating variants a more laborious and time-consuming task. While merely adding high entropy junk to each variant might appear as a low cost way to overcome this obstacle, it may backfire. The resulting variants will contain a high proportion of high entropy segments and will necessarily be larger. Their relatively high entropy may itself become suspicious, while their size may undermine their viability by hindering their propagation.

In our experiments, we found that the majority of false positives occurred in setup and installer files. These files perform actions that, from first principles, are similar to malware actions (e.g., change the file system or the registry). This result suggests that NCD might be able to capture behaviour typical of malware even across different malware families.

## 4. Evaluation

We designed our study to answer the following research questions:

RQ1: How accurate is NCD in classifying malware?
  As mentioned before, NCD is lightweight in that it can be applied directly to the binary executables without the need for reverse engineering or executing the programs. This research question investigates NCD’s performance (accuracy and false and true positive rates) in classifying programs as malware or benign-ware.
RQ2: How accurate is using compressibility rates in classifying malware?
  We expected malware to have higher entropy than benign-ware because, in the current state of the malware arms race, malware designers often use polymorphism (compression and encryption) to avoid detection. Therefore, we expected malware to be less compressible than benign-ware. Using compressibility rates is less computationally expensive than NCD because the compressibility rate is a feature of an individual program, while NCD is a pairwise feature. In this question, we investigated the performance of compressibility rates (accuracy and false and true positive rates) compared to NCD in classifying malware.
RQ3: Is malware reported in the same time frame more likely to have similar patterns?
  The malware we originally collected was reported by the public on the VirusWatch Archive in a period of eight consecutive days. This fact might influence the results if malware reported within a short time period is more homogeneous. We investigated this issue by repeating our experiments on a new sample set that was reported on randomly selected dates spread throughout a little under two years and examining the results.
RQ4: How much can we reduce the cost of using NCD by using approximations based on NCD lower bounds?
  The performance of an NCD-based classifier is expected to rely on the number of programs that are used to build and train the classifier. However, a larger number of samples leads to a larger number of NCD pairwise comparisons. Since the time complexity for NCD is quadratic, the effect of increasing the sample set on performance might make the approach impractical. We can use NCD lower bounds to approximate NCD and reduce the cost. However, before using approximations in the classifier, we first empirically quantified savings, in the number of computations, this approximation achieves in practice.
RQ5: How does an NCD classifier compare to commercial and open-source antivirus software?
  We labelled the programs in our samples as malware or benign-ware based on the source from which we obtained them. Although we expected this classification to be reasonably accurate, we could not guarantee that it was 100% accurate as benign-ware might be mistakenly reported as malware to the virus repository we used. Similarly, we could not guarantee that no malware existed in the benign-ware set. To have a ground truth, we needed to reverse engineer and analyse each program in the sample set; a labour intensive solution that is not practical. However, we could leverage antivirus software to gain a better understanding of our samples and our results.

### 4.1. Corpus

We collected malware and benign-ware to form the corpus for our experiments in the following way: For malware, we used a script to download all executable binaries automatically that were reported on the VirusWatch Archive http://lists.clean-mx.com/pipermail/viruswatch/ from the 4 until the 11 April. We configured the script to attempt to download a file a maximum of three times and to abort a connection after five seconds of idle time.

We also collected malware from the Kaggle malware competition https://www.kaggle.com/c/malware-classification. The dataset contained two subsets: train and test. Kaggle’s test data were not labelled, so we trained and tested on the train subset, formed by 10,869 malware files. The data instances were classified into nine malware families whose features are summarised in Table 1. There were two files per malware: a byte representation (hexdump) and an asm file with IDA Pro information from the disassembly process. We used xdd http://linux.about.com/library/cmd/blcmdl1_xxd.htm to convert the hexdumps to binary executables. This dataset was published February 2015.

For benign-ware, we collected all executables from two Windows 7 machines. We found collecting benign-ware to be more challenging than collecting malware because benign-ware sources do not offer executables, but rather offer installers and setup files. Collecting only installer files would not be representative of benign-ware, while using them to install applications and then collect the resulting binary files proved to be a laborious. Therefore, we decided to collect executables from existing machines.

After collecting all samples, we filtered the results to retain only Windows executables using the results of the Linux Fileutility. We then used the Linux fdupes utility to identify and remove any duplicate files. The fdupes utility compares files first by size, then the MD5 signature, and finally, uses a byte-by-byte comparison. Table 2 provides some statistics about our corpus.

To conduct our experiments and have multiple data points that could allow for statistical analysis, we randomly sampled 1000 programs from each type (malware and benign-ware) from our corpus. We repeated the sampling process (with replacement) three times, creating three sets of 2000 programs each. We then conducted each experiment in our study on each sample set independently and then analysed and compared the results. Statistics about the samples sets can also be found in Table 2.

### 4.2. Classifier Parametrisation

The random forest classifier we built to label programs as benign or malicious had a number of parameters that needed to be fixed before training and classification could take place.

The random forests we trained for all experiments consisted of 400 individual decision trees trained independently in parallel. Randomness was injected into the training process by considering a fresh random selection of 30 features (out of the available n+1) for training each tree branch, but making all training data available. Trees were grown, adding branches, until the information gain was less than 0.001 bits, or a depth of five branches was reached.

We experimented with training under two conditions: first, we trained the classifier with an equal number of benign and malicious training exampled; second, we severely biased the training set by using 10% benign and 90% malicious examples. The biased training condition heavily penalized false positives and should lead to fewer of those at the expense of increased false negatives.

### 4.3. NCD Classifier

**Figure 2:**
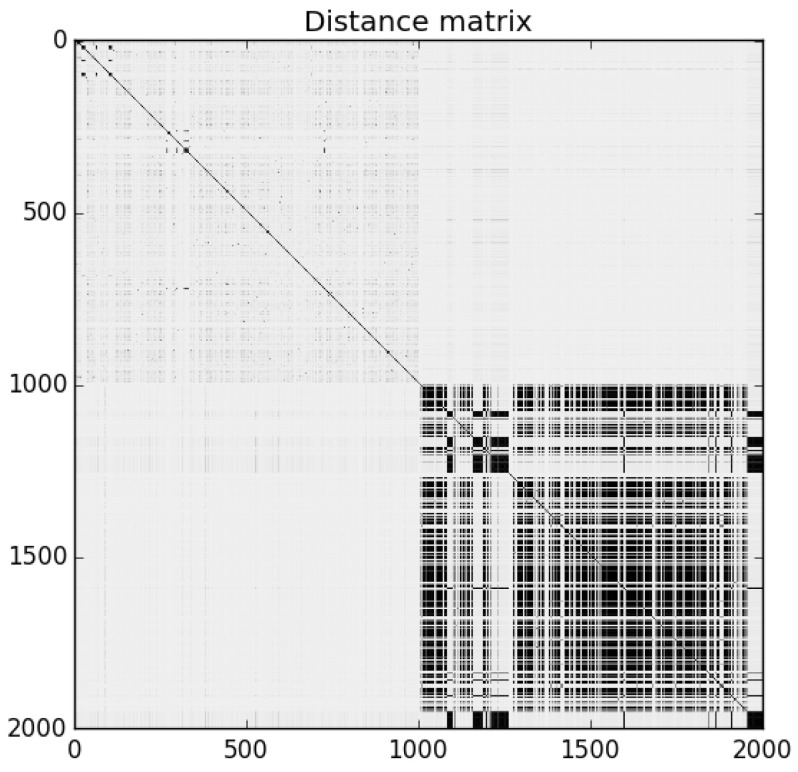
NCD matrix for Sample Set 1.

For each of our three 2000 program sample sets described in Section 4.1, we first calculated pairwise NCD using the 7-Zip compressor. Figure 2 is the distance matrix for Sample Set 1: the darker dots represent similarity (lower NCD), while lighter areas represent dissimilarity (higher NCD). The programs are ordered in the matrix such that benign-ware comes first, followed by malware. The matrix shows that the malware in the set was clearly homogeneous, while benign-ware was similar to neither other benign-ware nor malware. Similar results were observed for the other sample sets.

We applied the algorithm described in Section 3 to the three sample sets. We set the number of features to n=200+1, namely the compression ratio and the NCD from 200 randomly chosen programs: 100 known benign and 100 known malicious. The size of the training and test sets was 600 and 600, respectively (with 300 benign and malicious samples each). Because the results were dependent on the choice of subsets, we repeated the experiment 30 times for each set by randomly selecting different subsets to act as features, training and test sets. We note that the size of the sample gave limited resolution for very small true positive rates and false positive rates. This limitation could be overcome by using a larger (but more difficult to gather) corpus of malware.

**Figure 3:**
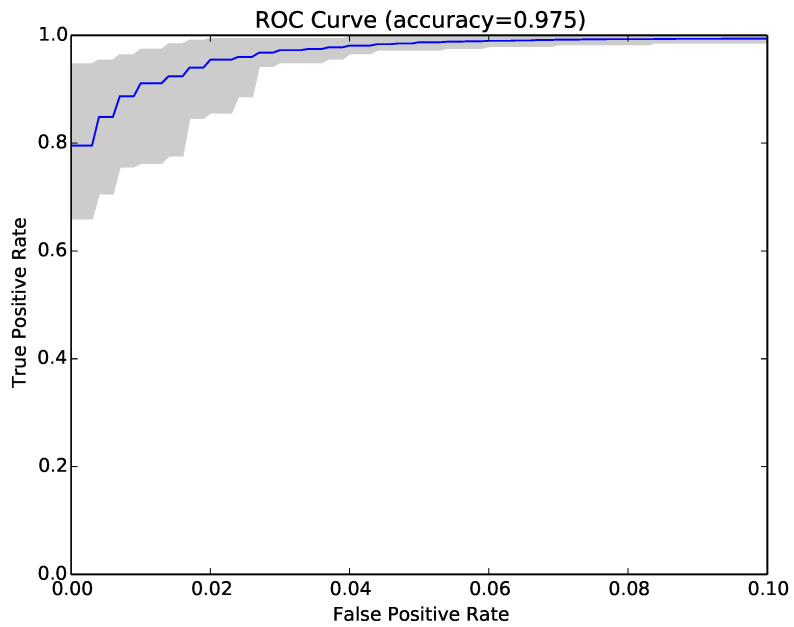
ROC curve for 30 runs of the NCD classifier for Sample Set 1.

**Figure 4:**
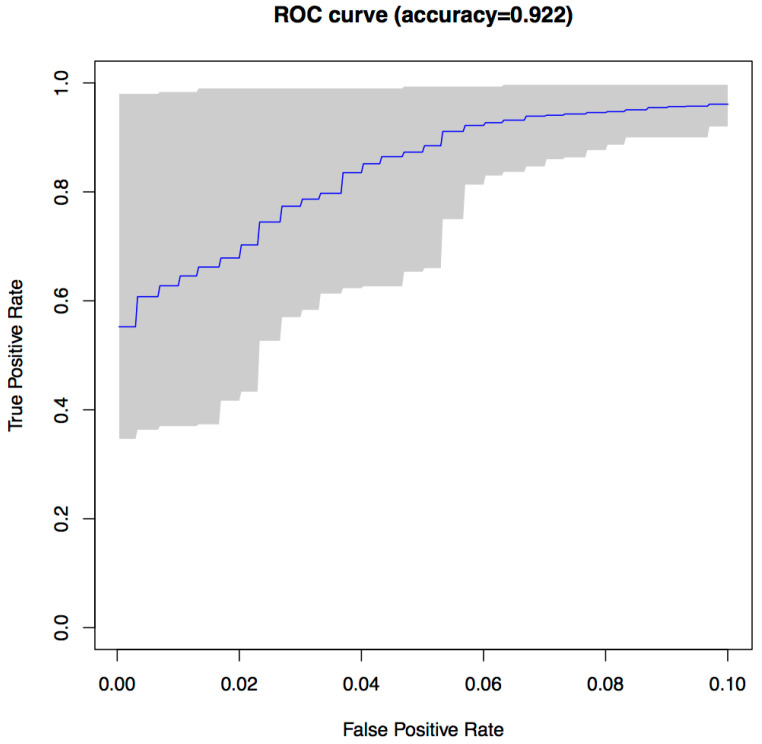
ROC curve for 30 runs of the NCD classifier for Kaggle Set 1.

The results for each run were represented as a Receiver Operating Characteristic (ROC) curve. Figure 3 shows the ROC curve for the 30 runs of the experiment for Sample Set 1, and Figure 4 shows the equivalent ROC curve for Kaggle Set 1. The blue curve shows the average results, while the grey area is between the maximum and minimum of the 30 runs. Each point on the curve represents the accuracy rates we could achieve by using a different vote threshold to differentiate between malware and benign-ware.

The x-axis is the false positive rate, while the y-axis is the true positive rate. The maximum accuracy that could be achieved across all runs (displayed at top of the graph) was 97.5% for Sample Set 1 and 92.2% for Kaggle Set 1. The average maximum accuracy achieved for Sample Set 1 was 97.1% with an average false positive rate of 3% and an average true positive rate of 97.3%. For Kaggle Set 1, the average maximum accuracy was 92.2% with an average false positive rate of 6% and an average true positive rate of 90.9%. However, if we chose a different threshold, we could reduce false positives or increase true positives by sacrificing accuracy. The choice of threshold depended on the objective we wanted to achieve. For example, in Sample Set 1, we could have no false positives if we chose a more conservative threshold, but accuracy and the true positive rate would be reduced, on average, to 92.2% and 84.9%, respectively. On the other hand, we could set the threshold higher and capture all true positives, but also capture on average around 22% false positives.

The results for the other samples were similar. The first set of columns of Table 3 (under header NCD) shows the average False Positive (FP), True Positive (TP), and Accuracy rates (Acc) for our experiments over 30 runs for each of the sample sets. The table also shows a comparison with the compressibility rate and structural entropy [9]. The last method extracted from the state-of-the-art was parametrised following the original work of Sorokin [9]. NCD outperformed both techniques in all cases.

In terms of time, the pairwise comparison was expensive for NCD due to the compression process. Table 4 shows the time comparison of NCD with other techniques. This problem could be significantly reduced if the compression steps were performed in parallel or the compression performance was reduced.

To understand how NCD and decision forests contributed to the observed results, we used a simple clustering algorithm (k-medoids) to cluster the programs in each sample set using NCD as the distance. In k-medoids clustering, *k* random points are selected as the centres (or medoids) of each cluster. All other data points are then assigned to the cluster in which they are closest to the medoid. A random point in the cluster is then swapped with the medoid, and the calculations are recomputed. If the swap causes the cost to be reduced (the sum of distances within each cluster), the new medoid is used; otherwise, the original medoid is kept. The process is repeated until there is no change in medoids. We applied this algorithm to each sample set setting *k* to 35, because from a grid of values ranging from three to 50, *k*=35 provided the best silhouette value, as it is usually applied in this context [10]. We labelled each cluster as a malware or a benign-ware cluster based on the dominating number of samples in the cluster. We then calculated false and true positive rates, as well as accuracy. The results (Table 5) showed that NCD alone could achieve good levels of accuracy in classifying malware and benign-ware (95.6–95.8% for the sample sets and 81.3–81.9% for the Kaggle sets); however, using decision forests improved the performance.

The answer to RQ1 is that NCD was effective at classifying malware and benign-ware and, when used in conjunction with decision trees, could achieve an average accuracy of 97.1% with average false positive rates of 3.2% and true positive rates of 97.6%.

### 4.4. Compressibility Rate Classifier

**Figure 5:**
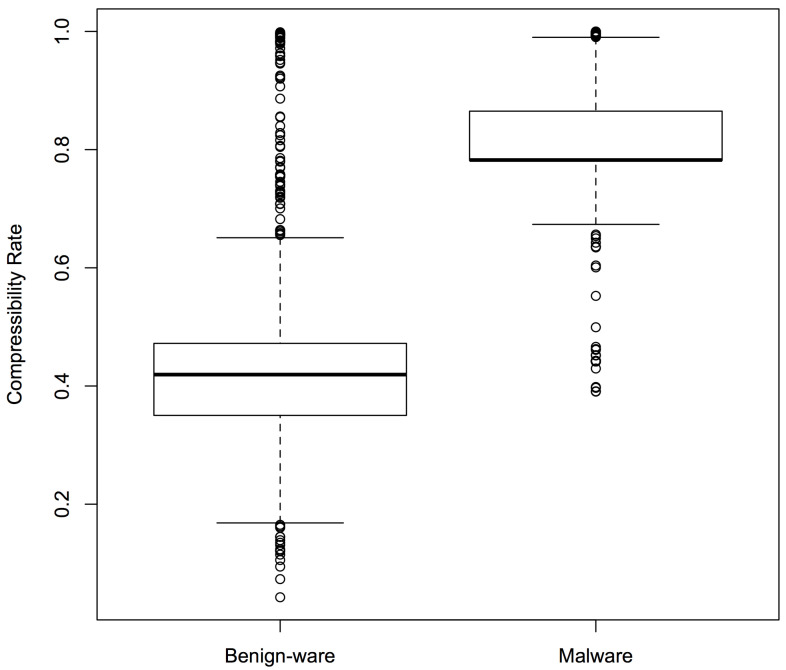
Compressibility rates of malware and benign-ware for Sample Set 1.

As mentioned before, we expected malware and benign-ware to have different compressibility rates (compressed size/original size). We plotted the compressibility rates of our samples to test our intuition. Figure 5 shows the box plots for Sample Set 1: the boxes represent the middle 50% compressibility rates of the sample, divided by the line that represents the median, while the whiskers represent the top (and bottom) 25%. The box plots confirmed that there was a clear difference in compressibility patterns between malware and benign-ware. Similar results were observed for the other two sets.

**Figure 6:**
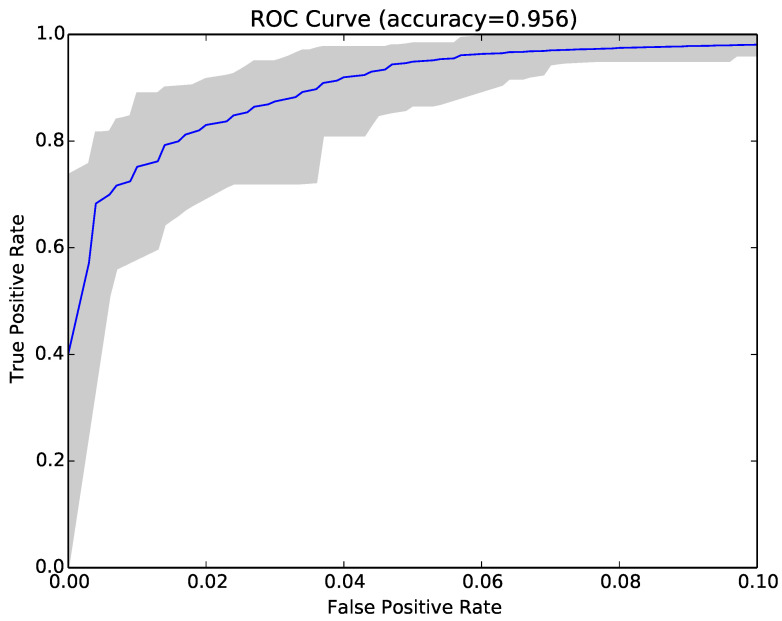
ROC curve for the compressibility rate classifier over 30 runs for Sample Set 1.

Inspired by these encouraging results, we repeated the experiments in RQ1 using compressibility rates as features instead of NCD. The main benefit of using Compressibility Rates (CR) instead of NCD was that NCD is a pairwise calculation, while CR is just calculated once for each program. The results (second set of columns in Table 3) showed that the accuracy achieved with CR was lower than that obtained by NCD, and the reduction was on average around 1.5% (97.1–95.6%) for sample sets and 15.8% (91.5–75.7%) for Kaggle sets. It was also lower than the accuracy of structural entropy. However, the time consumption comparison in Table 4 shows that this technique was the fastest one. Figure 6 shows the ROC curve (blue curve) and variation (grey area) in false positive and true positive rates using the compressibility rate classifier with the same sample of programs as Figure 3.

**Figure 7:**
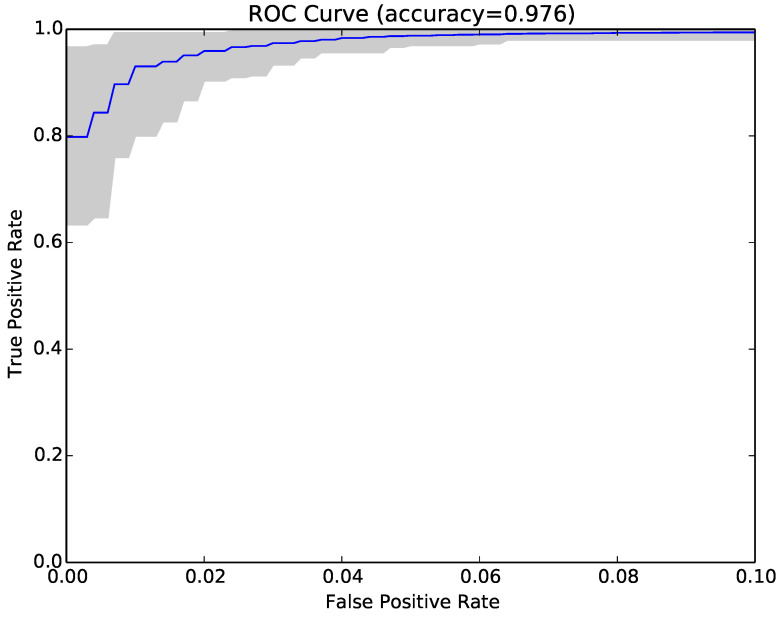
ROC curve for 30 runs of the combined compressibility rate and NCD classifier for Sample Set 1.

Finally, we tried combining NCD with compressibility rates to see if the two approaches were complementary. The combined approach achieved on average higher accuracy (97.4% vs. 97.1% and 91.9% vs. 91.5%), lower false positive rates (3% vs. 3.2% and 5.7% vs. 5.9%) and higher true positive rates (97.9% vs. 97.6% and 89.5% vs. 89%). The average results over 30 runs are reported in the last set of columns in Table 3), while Figure 7 shows the ROC curve for the same sample as Figure 3 and Figure 6.

We applied a two-sided Mann–Whitney test to the accuracy observations and found that the differences between the three approaches were statistically significant with 95% confidence.

The answer to RQ2 is that the compressibility rates were effective in classifying malware and benign-ware and could achieve an average maximum accuracy of 95.6% with average false positive rates of 5.1% and true positive rates of 96.4%. NCD classifiers performed statistically significantly better than compressibility rate classifiers. Combining NCD and compressibility rates statistically significantly improved performance.

### 4.5. Size of Malware Reporting Window

A threat to the validity of our study was that the malware we collected was reported within a short time frame. If malware spreads in outbreaks where several variants are released by an adversary at the same time, this might suggest that our approach could lose effectiveness over time. To investigate this issue, we repeated all our experiments with samples that had more diverse reporting times. We selected 10 random dates from the period between 1/1/2013 and 1/10/2014. We then downloaded all the Windows executable malware reported on those 10 dates and processed them in a similar manner to our original corpus. We then randomly selected 1000 malware from this new corpus and 1000 benign-ware from our original set of benign-ware and repeated the experiments in RQ1 and RQ2.

**Figure 8:**
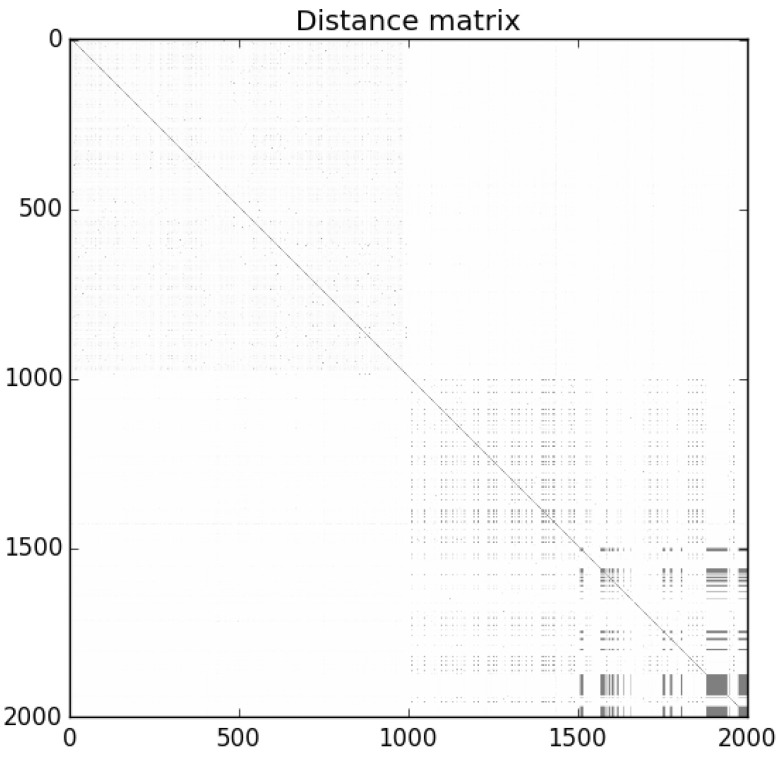
NCD matrix for a diverse (with regards to reporting time) sample set.

Figure 8 shows the distance matrix for the new diverse set. The malware in this sample was noticeably less homogeneous than the previous sets. However, the same general conclusion still held: malware was more similar to malware than benign-ware while benign-ware was not similar to other benign-ware or malware.

**Figure 9:**
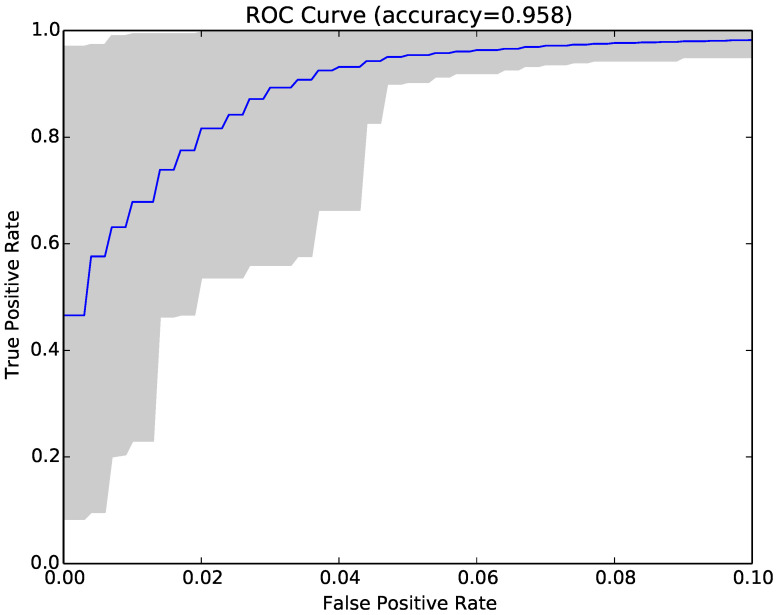
ROC curve for 30 runs of the NCD classifier for the diverse sample set.

**Figure 10:**
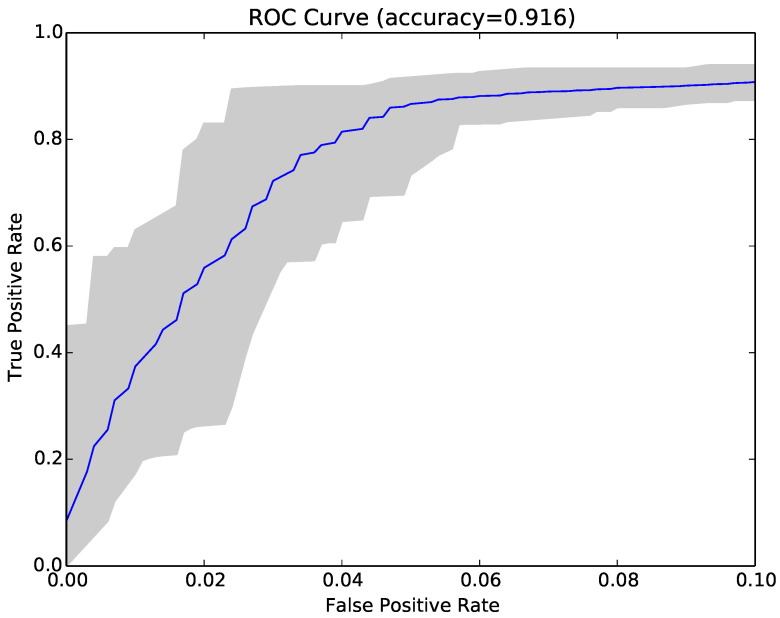
ROC curve for 30 runs of the compressibility rate classifier for the diverse sample set.

**Figure 11:**
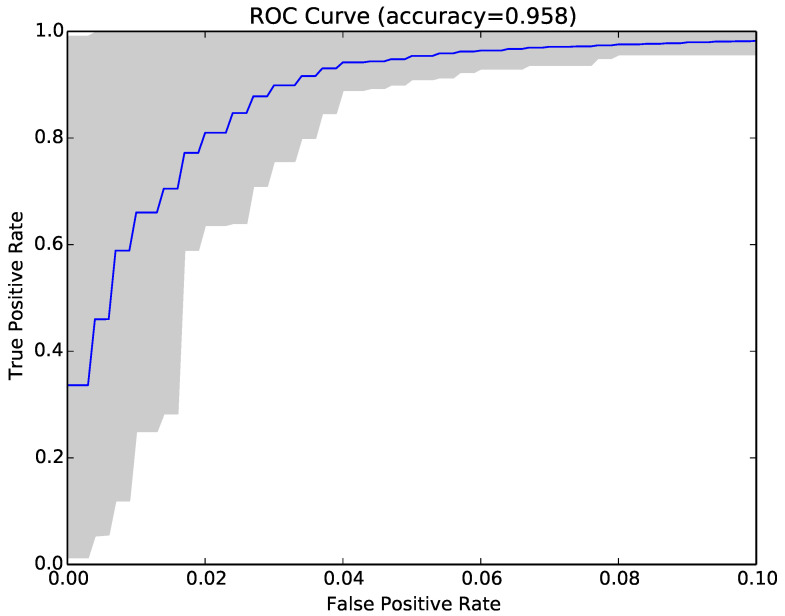
ROC curve for 30 runs of the combined compressibility rate and NCD classifier for the diverse sample set.

Table 6 depicts the average best accuracy across 30 runs of the classifier for NCD, compressibility rates, and the combined approach. Figure 9, Figure 10 and Figure 11 show the ROC curves for our 30 runs for the NCD classifier, the compressibility rate classifier, and the combined classifier. The overall average accuracy results for each approach were slightly lower than those obtained from the previous samples. Interestingly, the results in these figures contained higher variation, as one can observe in the larger grey area in each graph, compared to Figure 3, Figure 6 and Figure 7. This higher variance was expected; it was a consequence of the greater malware diversity of this dataset. Further, this variance and the telltale staircase pattern of low resolution in the ROC curves were confined to FPR below 5% (due to the size of our sample sets, Section 4.3), but disappeared above that threshold.

Inference always depends on the choice of feature and training sets; this result suggested that, for our approach to be successful in practice, we may need to evolve the classifier over time. This might not be a significant concern since the process of building the classifier was completely automated and safe (since it did not require running the malware). This result also suggested that rebuilding the classifier need not be frequent since, even across a two year interval, the classifier was still reasonably accurate.

**Figure 12:**
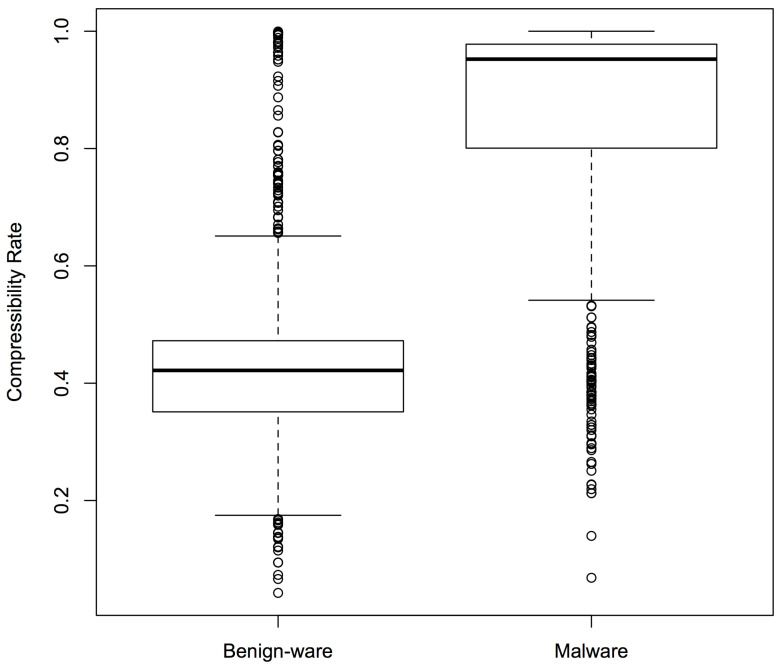
Compressibility rates of malware and benign-ware for the diverse sample set.

**Figure 13:**
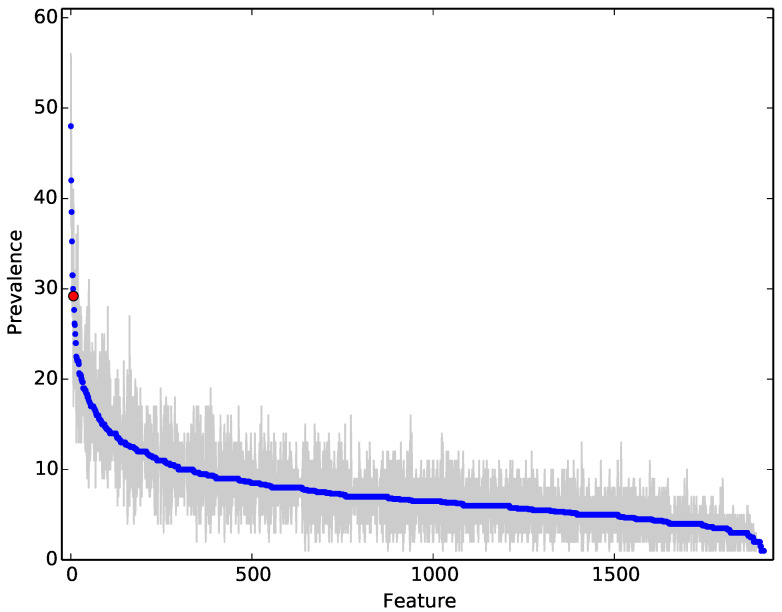
Relative feature importance.

Figure 12 shows the difference in compressibility rates between malware and benign-ware for the diverse set. Similar to the previous sets, there was a clear difference between the two sets. However, the average accuracy was much lower than the accuracy observed for the previous sets. This might be caused by the larger number of outliers in malware that had a lower compressibility rate. Figure 13, which is representative of the importance gain of our experimental runs, suggests that our data contained “Kevin Bacon” programs that effectively partitioned the rest of the programs because they lay near, and even defined, the centre of clusters in the data. In the upper left of the Figure 13, these programs gave rise to the NCD measures that were the most prevalent feature. Thus, another reason for the drop in our accuracy may be due to the fact that the more diverse a dataset was, the less likely one was to select these highly discriminant, “Kevin Bacons”.

The answer to RQ3 is that malware reported in a tighter time frame was more homogeneous than malware reported over a longer period of time. Nonetheless, our NCD classifier still achieved 95.2% accuracy with 5% false positive and 95.4% true positive rates, on average.

### 4.6. NCD Cost Reduction

As mentioned before, we could reduce the cost of computing NCD in terms of time and computation power by skipping calculations that we thought might not contribute to the effectiveness of the classifier. For example, we might decide that if the lower bound on NCD for a pair of programs was 0.99, the effort needed to compute the real value (which would be between 0.99 and 1) was not justified.

**Figure 14:**
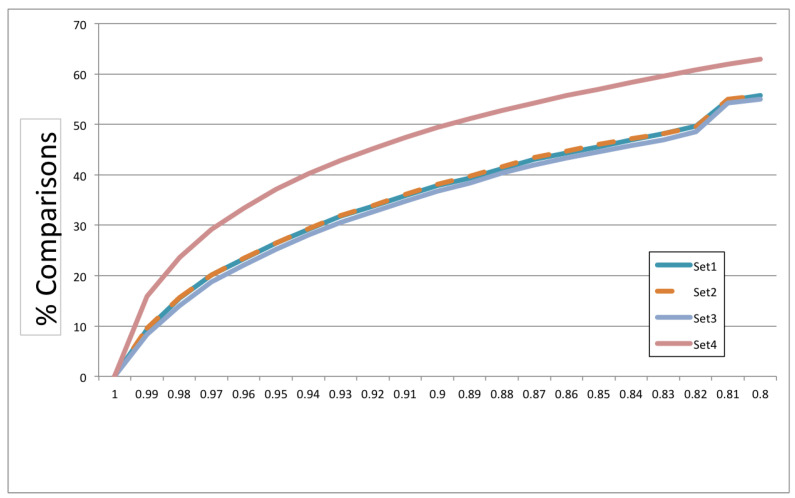
Savings in percentage of NCD calculations using lower bound thresholds.

We empirically estimated the percentage of calculations that we could skip by using lower bounds on NCD by setting the threshold at different values. For each of our four sets, we counted the number of pairs where the lower bound on NCD was equal to or above a number of thresholds that ranged from 0.8 to one with 0.01 increments. Figure 14 shows the plot for each set. The x-axis is the different thresholds of NCD, while the y-axis is the saving in percentage of computations. Each set consisted of 2000 programs; therefore, the total number of computations needed was 2,001,000.

The graph shows that the results for the three original sets were almost identical, while the diverse set offered even more savings at each point of the threshold. If we set the NCD lower bound threshold at which we relied on approximation to 0.99, we already saved 8–16% in the number of calculations. The percentage of calculations we could save grew gradually with the threshold and reached between 37 and 49% even at a threshold as high as 0.9. These results indicated that we could reduce the cost of using NCD by using this simple technique of approximating lower bounds. Approximating NCD lower bounds could be considered a more refined way to use compressibility rates to classify, since these lower bounds compared the compressed size of two strings individually rather than the compression of their concatenation.

The answer to RQ4 is that we could achieve savings from 8–16% by setting the lower bound threshold to use approximations to 0.99. The savings we could achieve increased as we reduced the threshold and reached 55–63% for a 0.8 threshold.

### 4.7. Comparison to Antivirus Software

To gain a better understanding of our results, we scanned all samples using Virus Total. Virus Total https://www.virustotal.com (a subsidiary of Google) provides an online service that scans files using up to 59 different antivirus engines. The service also provides an API that can be used in batch scanning. We used uirusu https://github.com/arxopia/uirusu, which is an interface written in Ruby to simplify uploading and scanning files using the Virus Total API.

The Virus Total website only accepts files smaller in size than 64MB, while the API only accepts files smaller than 32 MB. It was sufficient to submit the MD5 hash code of a program if the file itself was submitted and analysed previously by another user. We scanned our malware and benign-ware samples using the API. Naturally, because we had no ground truth, we could only report on false positives and negatives by assuming that our initial labelling was correct. However, the results of scanning might give us an insight into our results and our samples. For this experiment, the Kaggle malware was not submitted, due to the original PEheaders being removed from the dataset, and consequently, the antivirus was not able to run the malware.

Table 7 summarises the results of the Virus Total scan on Sample Set 1 and the diverse set (Sets 2 and 3 showed similar results to Set 1). The first row shows the number of scanned files. All programs were scanned successfully except six malware programs from Sample Set 1 and 20 from the diverse set, which violated the size limitation. The number of programs classified by at least one antivirus engine as malware is shown on the second row. Interestingly, the number of malware detected in Sample Set 1 was considerably higher than the number detected for the diverse set (941 vs. 789). This mirrored our NCD classifier results where the average best accuracy across 30 runs for Sample Set 1 was higher than that for the diverse set (97.1% vs. 95.2%). This result might indicate that the difference in NCD classifier accuracy for the two sets was caused by inaccuracy in the original labelling rather than the diversity of the sample set. However, more experiments have to be conducted to verify this observation.

We also noticed that in both sets, around 10% (94 and 105) of benign-ware was classified by at least one antivirus engine as malware. Examining the names of these misclassified programs revealed that they were mostly uninstallers, setup files, and updaters. Interestingly, we observed a similar phenomenon in our NCD classifier. The similarity between malware and uninstallers that was detected by NCD might be caused by the fact that they perform similar actions (e.g., writing to the file system, changing the registry). This might suggest that some of the antivirus engines used in Virus Total work in a similar manner to NCD (i.e., identify similar patterns). However, a more detailed analysis is needed to determine if these detected files are really false positives or if they are in fact malware. It is also worth noting that the majority of these programs were only flagged as malware by one or two engines out of the 59 engines used in Virus Total. In fact, only 11 programs out of all benign-ware were classified as malware by more than five engines. The engine that flagged the most benign-ware as malware for both sets (45 and 47) was only able to detect a small number of malware (35 and 54) in Sample Set 1 and the diverse set. This engine claims to use a DNA matching algorithm to detect malware, which might explain the similarity in false positives to our NCD classifier.

The final two rows of the table show the highest and lowest number of malware and benign-ware that were classified by a single antivirus engine as malware for each sample set. Again, we could see a noticeable difference between the two sample sets in the number of detected malware. The best performing engine in Sample Set 1 found 883 out of 994 malware (88.8%), while in the diverse set, the best performing engine found 525 out of 980 (53.6%). In each set, a different engine had the best performance. Table 8 shows the false positive, true positive, and accuracy rates for these two engines. Engine 1 was the best performing engine in Sample Set 1 and the second best performing engine in the diverse set. We could see from the results in the table that the false positive rate was around 2%; however, accuracy rates were considerably lower than those obtained by any of our classifiers.

Engine 2 was the best performing engine in the diverse set, but only detected 175 malware in Sample Set 1. This engine had a very low false positive rate, but also suffered from low accuracy. Note that we could achieve low false positive rates in our classifiers by adjusting the voting threshold in the decision forest while achieving higher accuracy rates than those achieved by these two engines. In both samples, our classifiers achieved considerably higher true positive rates than the combined performance of all 59 antivirus engines. We also had a lower false positive rate and higher accuracy. These results, however, were provided for reference and could not be used to compare our approach to these engines. The reason was that these results were for the whole set, while our approach used a subset of programs as feature and training sets. Additionally, as we explained before, we did not have a ground truth, and we did not know how these commercial engines worked and how much manual effort was involved in building them compared to our approach, which was fully automated.

### 4.8. Feature Importance Analysis

We followed the methodology by Breiman [7] to evaluate the importance of specific features, on the basis of how often they were used in the random forest classifier. Figure 13 illustrates the relative importance of each feature averaged over 30 independent experiments (a new split of training vs. test data and a fresh forest of 100 trees trained). The grey area denotes the min-max values for each feature and the red dot the feature corresponding to compressibility ratio. Thus, this graph illustrates that while the compressibility feature was relatively informative, it was not the most informative one (which are and why are interesting questions). The features are ordered by importance on the x-axis, with the most used features to the left and the least used to the right. On the y-axis, we plot the prevalence, a proxy for importance, of each feature within the random forest.

It is noteworthy that a bag of about 10–20 features is relatively important and used quite often. By inspection, we noted that compressibility was usually within these commonly used features, but was not always the most common one. This illustrated, along with the improved performance, that NCD did add value to classification and provided valuable features. We also noted that the bulk of features provide some value, and we conjectured the keeping a varied portfolio made classification more robust and general.

## 5. Related Work

Since NCD has been around since 2003, it is not surprising that there are a number of papers that use or refer to Kolmogorov complexity or information theoretic techniques, not necessarily NCD. These can be grouped into two closely related, but different problems: those that consider detection only (as does this paper), and those that consider classification, either in conjunction with the detection problem or by itself.

### 5.1. Detection

The first works based on statistical malware detection were based on n-gram analysis. For instance, Kephart’s work [11] focused on an n-gram approach for extracting signatures. From the machine learning perspective, one of the first approaches was introduced by Schultz et al., who aimed to distinguish between benign-ware and malware in Windows or MS-DOS format, by combining machine learning and n-grams. Their bottleneck was the scalability related to the value of n. They used a corpus of 3265 malware and 1001 benign-ware, reaching 97.11% of accuracy with a 3.8% false positive rate [12].

An interesting approach based on information gain and n-grams was introduced by Kolter and Maloof. Their experiments combined two different corpora. The first contained 476 malware and 561 benign-ware. Their validation results in this corpus were 95% accuracy with a 5% false positive rate. The second corpus contained 1971 benign-ware and 1651 malware. Their validation results were 94% accuracy and a 1% false positive rate [13]. At the same time, Stolfo et al. developed an n-gram approach that they compared with different file type models for malware detection within DOCand PDF files. Their evaluation used 361 benign-ware and 616 malware files. The results of this evaluation were between a 3% and 20% false positive rate, but the authors reported no accuracy [14].

Apart from the previous works based on n-grams, there were several works based on entropy. For instance, Lyda and Hamrock used the average entropy to show that high entropy regions in binary files correlated with encryption or compression. Although they evaluated this hypothesis in a corpus of 20,000 malware, they did not focus their experiments on malware detection [15].

Hybrid methods like the one introduced by Santos et al. aim to reduce the labelling process. This work used a semi-supervised methodology based on n-grams. Their evaluation was performed on 1000 malware and benign-ware, achieving 89% accuracy with 10% false positives [16].

Dang, Liu, and others used Dynamic Markov Compression (DMC) to classify binary executables as malware or benign-ware. Neither their method, nor their results were reported in a clear way [17]. They did some light pre-processing by removing headers and white spaces and converting each executable to hexadecimal. For each executable, they selected substrings of length 1 KB, 10 KB, and 100 KB in a manner not explained. This was to reduce the space overhead required in their compression approach. They compressed candidate strings using DMC models for both known benign-ware and a known set of malware. The candidate was classified according to the smallest compression of the file achieved. They experimented on 2000 benign-ware and 1000 malware with 15% of the benign-ware used for training along with 30% of the malware and the remainder in a test set. The results were given as ROC curves, but without any discussion, accuracy rates, or other information.

Gong, Tan, and Zhu devised a detection scenario for malware through the use of compression with Prediction by Partial Matching (PPM) [18]. They built Markov models of different malware families, but only offered a “preliminary experiment”. This seemed to consist of 200 malware divided into a training and a test set in a 30%:70% split. However, they did not provide any results nor descriptions of the experiments.

Abbas and Harris sketched an intrusion detection system that employed compression and hashing [19]. Their system had components that profiled worms, profiled the network, profiled malware, and profiled events on the system. All of these profilers employed NCD. The malware profiler was intended to test samples gathered from various sources against various antivirus vendors to establish some ground truth. Then, the sample, once identified as malware, was run in a sandbox environment, and information was collected. A fuzzy hash was created that acted as a profile. A matrix of NCD values was created for the set of profiles and used to determine a distance threshold, which was saved with the database. Collectively, this became the malware detector. The report of the “initial runs” of the system was not clear with regard to success in malware detection.

Windows malware analysis aiming to integrate dynamic and static analysis, as [20,21,22], to produce features for data mining approaches suffer the same problems.

### 5.2. Classification

Wehner used NCD to cluster worms and compressibility to identify packed and encrypted traffic on a network [3]. She used rootless tree diagrams as per the CompLearn tool developed by Cilibrasi [23] together with a discussion of the clustering results. There was no rigorous experimental approach or intention to evaluate NCD as a classifier.

Bailey, Overhead, and others used NCD in the context of clustering data derived from five minutes of instrumented execution of malware on a virtual machine [24]. Their aim was to address the inconsistencies and variations that arose in labels for malware programs from the various tool specific systems. They argued that a label system for malware files should be consistent and complete and that labels should be concise. They demonstrated that existing systems failed to achieve this by assembling a corpus of 3700 malware collected between 2004 and 2007 and analysing these files with five different AV tools. They used the Backtracker system to capture event information during the execution. After extracting information of interest, such as files modified, processes created, and network connections made, they used this to create an execution profile for each program. By applying NCD pairwise to the profiles, they had the raw material for a hierarchical clustering algorithm, and they evaluated the results of this with respect to the consistency, completeness and concision of the labels in comparison to those produced by the malware tools. Although their experiments were comparable to ours in scale, they did not aim to detect and classify programs as malware or benign-ware.

Apel, Bockermann, and Meier experimentally evaluated a set of distance measures for programs with the aim of identifying the most appropriate measure for clustering malware based on behaviours [25]. They considered this as contributing to a detection approach, but their experiments only sought to cluster malware rather than classify candidates. They collected 1195 malware samples from honeypot sites and produced traces from these using CWSandbox. They complained about the difficulty of establishing some ground truth with respect to the files. They used the traces to compare and evaluate four distance metrics: Levenshtein distance, approximated edit distance, normalised compression distance, and Manhattan distance using tries. They ranked these distances according to their ability to cluster the traces, measured by the number of continuous (sic) system call sequences that were shared by the traces in the cluster. Their conclusions included that NCD should not be used to analyse malware execution traces.

Gurrutxaga, Arbelaitz et al. also evaluated a set of distance measures for their suitability for clustering dynamic traces produced by malware [26]. They cited both Bailey et al. and Wehner. Malware was executed for one minute in a controlled environment, and information about the execution was collected. They compared different metrics, including NCD, as well as two different ways of representing the collected data. They used three hierarchical clustering algorithms and evaluated each of these three sets of choices

Wicherski developed a fast, non-cryptographic hashing algorithm for malware clustering that worked on files in Portable Execution format [27]. The information hashed included the file’s compressibility ratio as an upper approximation to its Kolmogorov complexity, alongside structural properties such as heap commit size. Again, the aim was to cluster malware rather than develop a detector. He tested his algorithm on two corpora consisting of 184,538 and 90,105 malware and evaluated the resultant clusters by examining the names of the malware and by comparison with clustering on benign-ware where he could more easily obtain a ground truth.

Calliat, Desnos, and Erra speculated that NCD might be used as a first filtering tactic to select malware from a database of known malware to find those most similar to an unknown malware [28]. Their paper offered no experimental evidence, and it was not clear whether they were aiming to detect or only classify.

Although it was an application neither of NCD, nor of compression, Baysa, Low, and Stamp’s investigation of similarity between malware groups produced by metamorphic engines was interesting as a point of comparison as it was entropy based and applied directly to executables [29]. Their similarity measure relied on earlier work by Sorokin, who developed a comparison technique that compared (executable) files via what he named structural entropy [9]. This involved using wavelet analysis to split each file into segments of varying entropy levels. Bays et al. used structural entropy comparison as a filtering method, then computed a similarity score via the Levenshtein distance between corresponding segments for files that passed the filter. They applied their similarity measure to three groups of malware files, each group produced by applications of a single metamorphic engine, and investigated how well the similarity measure distinguished the group from a fixed group of 16 benign-ware. They reported 100% detection accuracy for the malware groups produced by the G2and MWORengines and an accuracy of 0.93539 for the detecting the group produced by the NGVCKengine.

Finally, Symantec’s AESOPdetection system is an interesting commercial example of leveraging an existing detection and classification tool (in this case, Symantec’s) via a similarity measure to enable detection on a larger scale while avoiding the need to analyse every file statically or dynamically [30]. AESOP relies on the large database owned by Symantec and voluntarily contributed to by users of Norton utilities (Norton Community Watch). Through the simple observation that some machines/users tend to accumulate malware, while others do not, they can perform a highly lightweight analysis of file associations via machines and, starting from the ground truth established by their antivirus tool, classify billions of files within realistic time scales. NCD/compression is computationally expensive, but requires no proprietary database of millions of machines and their files. Furthermore, scalability can be radically improved through the use of a classifier.

### 5.3. Other Approaches

Malware is constantly evolving and adapting to new platforms. A good example is the new amount of Android malware in the wild. Since the introduction of Drebin [31], several authors have focused their efforts on Android malware detection and classification [32,33]. Furthermore, new tools have increased the analysis capabilities in this area. Good examples are CopperDroid [30], a behavioural analysis tool focused on dynamic analysis, and DroidSIFT [34], an anomaly detection tool focused on malware family classification.

Similarly, other tools employ network analysis for malware detection based on graph theory, for example Nazca [35] and AESOP [36]. These tools show that they can improve the scalability of the detection process keeping a good compromise with the detection accuracy. Other tools focus on malware propagation, such as the work of Zhongqiang Chen et al., who detected malware based on propagation patterns [37]. Current tools follow this philosophy of understanding malware behaviour and aim to execute all the possible behaviours of the malware. A good example is the work of Sebastio et al., who stressed different malware behaviours via symbolic execution [38].

Modern approaches measure the limitations of machine learning during the detection process. These techniques form the field of adversarial machine learning. This field exploits the vulnerabilities of a learning system [39] to produce a misclassification. The adversary manipulates the data instances in a semantically equivalent way. The new samples produced, or variants, aim to be classified in a different class than the original malware. These ideas were originally studied on spam detectors [40].

EvadeML, an adversarial tool against pdf malware detection, was the first adversarial method applied in the malware context [41]. This tool defeated two machine learning-based detectors from the state-of-the-art: Hidost [42] and PDFrate [43]. Using information from the features of pdf files, EvadeML injects benign information to alter the feature distribution. Similar approaches, like EEE [44], extended this idea to Windows malware combining intelligent packing and evolutionary computation to create the malware variants. These approaches have been applied to the family classification problem, as well. For instance, Calleja et al. [45] applied adversarial machine learning to cheat malware family classification based on static analysis. These ideas have also been extended to audit antiviruses by measuring the way they can be mimicked [46].

A good compilation of different adversarial machine learning techniques can be found in the work of Biggio et al., who studied these effects from support vector machines [47] to different adversarial models in the wild [48].

## 6. Conclusions

We demonstrated with statistical rigour that NCD can be used to detect malware in the form of disk-resident executable Windows binaries. Since we used a similarity metric applied directly to the executables, we were essentially leveraging any available ground truth represented by an existing zoo of malware and benign-ware, in a completely automated way without any need for static or dynamic analyses. It goes with out saying that our approach did not obviate the need for analysis to establish a ground truth.

We showed that our approach competed very well indeed with existing malware detection programs and was as good as any of them when applied to our data. The genericity of NCD implied that our approach could be applied to many other string types useful in malware detection and classification, for instance source code such as JavaScript, execution trace information, and system logs. The approximation to universality of NCD means that it is difficult for malware writers to get a handle on techniques to evade detection via it. By combining compressibility rates with NCD in the decision forest classifier, we showed that it was possible to use the classifier as a combiner of metrics to make incremental improvements in the accuracy rate.

We plan on experimenting with selecting those features that provide the most information (“Kevin Bacons”) and building less expensive classifiers on this basis. Similarly, we will deeply study the benefits of NCD for clustering methods to reduce the labelling effort of malware analysers. Another possibility for enhancing the scalability of the approach is investigating the Nyström method as a way to interpolate internal values of NCD distance matrices [49]. Just as for the bounds method discussed in Section 4.6, reducing the number of features, and estimating matrix entries may make classification faster, but at the possible cost of reducing robustness and accuracy. Establishing the optimal trade-off between accuracy and performance is a promising avenue for future work.

## Figures and Tables

**Table 1:** Information about Kaggle classes: number of instances, concealment strategy, and type of malware.

Class	Instances	Conc.	Type
Ramnit	1541	Poly	Worm
Lollipop	2478	Poly	Adware
Kelihos_3	2942	Poly	Botnet
Vundo	475	Meta	Trojan
Simda	42	Poly	Botnet
Tracur	751	Poly	Trojan
Kelihos_1	398	Encr	Botnet
Obf.ACY	1228	Meta	Trojan
Gatak	1013	Poly	Trojan
Total	10,868	10,000	3703

**Table 2:** Descriptive statistics of the malware and benign-ware samples used in our study.

	All Samples			Set 1		Set 2		Set 3	
		Size (KB)		Size (KB)		Size (KB)		Size (KB)	
Type	No.	Mean	Med.	Mean	Med.	Mean	Med.	Mean	Med.
Benign	3046	932	116	903	118	766	112	1006	110
Malware	14,656	4287	500	3120	500	3279	501	2449	501
Kaggle	10,869	1025	1008	1062	1256	1021	1210	1028	952

**Table 3:** Average best value for accuracy with average corresponding false positive and true positive rates for each sample set and each approach.

Sample	NCD			Compressibility Rate			Combined			Structural Entropy		
	FP	TP	Acc	FP	TP	Acc	FP	TP	Acc	FP	TP	Acc
Set 1	0.030	0.973	0.971	0.057	0.961	0.952	0.030	0.974	0.972	0.150	0.800	0.824
Set 2	0.034	0.978	0.972	0.037	0.961	0.962	0.030	0.985	0.977	0.150	0.750	0.806
Set 3	0.034	0.976	0.971	0.060	0.969	0.955	0.030	0.978	0.974	0.150	0.820	0.828
All	0.032	0.976	0.971	0.051	0.964	0.956	0.030	0.979	0.974	0.150	0.790	0.819
Kag1	0.064	0.909	0.922	0.232	0.705	0.737	0.063	0.897	0.917	0.150	0.900	0.833
Kag 2	0.051	0.865	0.907	0.230	0.761	0.765	0.051	0.874	0.911	0.150	0.890	0.889
Kag 3	0.063	0.895	0.916	0.216	0.755	0.770	0.057	0.915	0.929	0.150	0.930	0.902
All	0.059	0.890	0.915	0.226	0.740	0.757	0.057	0.895	0.919	0.150	0.907	0.875

**Table 4:** Time consumption values for the experiments using the different techniques: NCD, compressibility rate, and structural entropy.

Sample	NCD	CompRate	Structural Entropy
Set 1	120 m	20 m	55 m
Set 2	121 m	19 m	54 m
Set 3	116 m	19 m	56 m
Kag 1	301 m	31 m	79 m
Kag 2	297 m	29 m	79 m
Kag 3	299 m	31 m	80 m

**Table 5:** Accuracy, false positive, and true positive rates for each sample set using NCD and k-medoids clustering with 35 clusters.

Sample	FP	TP	Acc
Set 1	0.052	0.968	0.958
Set 2	0.040	0.952	0.956
Set 3	0.042	0.953	0.956
All	0.045	0.958	0.957
Kag 1	0.273	0.910	0.819
Kag 2	0.217	0.962	0.818
Kag 3	0.327	0.843	0.813
All	0.272	0.905	0.816

**Table 6:** Average best value for accuracy with average corresponding false positive and true positive rates for the diverse sample set and each approach.

Approach	FP	TP	Acc
NCD	0.050	0.954	0.952
Comp Rate	0.057	0.879	0.911
Combined	0.057	0.962	0.953

**Table 7:** Summary of the results of scanning Sample Set 1 and the diverse set using Google’s Virus Total service.

	Set 1		Diverse Set	
	Mal.	Ben.	Mal.	Ben.
Scanned	994	1000	980	1000
Detected	941	94	789	105
Not Detected	59	906	206	895
AV Highest	883	45	525	47
AV Lowest	0	0	0	0

**Table 8:** Scan results of the top performing engines in both sets.

	Set 1			Diverse Set		
	FP	TP	Acc.	FP	TP	Acc.
Engine 1	0.019	0.888	0.935	0.022	0.529	0.756
Engine 2	0.006	0.176	0.586	0.005	0.536	0.768
All	0.094	0.947	0.926	0.105	0.805	0.851
